# Dr. James Read Chadwick

**Published:** 1905-10

**Authors:** 


Dr. James Read Chadwick, of Boston, was found dead at his summer home, Chocoma, N. H., September 24, 1905, aged 60 years. The presumption obtained that, becoming ill in the night, he sought fresh air on the roof of the veranda, fainted or lost his balance and fell to the ground, causing instant death. Dr. Chadwick, in association with the late Dr. Paul F. Mundé, founded the American Gynecological Society, these two being entitled to be called "sole founders" of that distinguished society. He was prominent in literature and everything that served for the advancement of medical science.

